# CBCT evaluation of multiple idiopathic internal resorptions in permanent molars: case report

**DOI:** 10.1186/1472-6831-14-39

**Published:** 2014-04-16

**Authors:** Atakan Kalender, Meltem D Öztan, Fatma Basmaci, Umut Aksoy, Kaan Orhan

**Affiliations:** 1 Department of Endodontics, Faculty of Dentistry, Near East University, 90392, Nicosia Cyprus, Mersin 10, Turkey; 2 Department of Endodontics, Faculty of Dentistry, Ankara University, Ankara, Turkey; 3 Department of Dentomaxillofacial Radiology, Faculty of Dentistry, Near East University, Mersin 10, Turkey

**Keywords:** Cone beam computed tomography, Endodontics, Idiopathic, Multiple internal resorption, Pulp inflammation

## Abstract

**Background:**

Internal inflammatory root resorption is a rare condition in permanent teeth, which requires the presence of necrotic and infected pulp tissue within the coronal portion of the root canal system as well as inflamed pulp tissue apical to the resorptive defect. The aetiology of internal root resorption is not completely understandable, trauma and chronic pulpitis are considered the main risk factors.

**Case presentation:**

We report a rare case of the multiple idiopathic resorption in the permanent maxillary and mandibular molars in a healthy 33-year-old female patient. In addition to clinical examination the patient was imaged using conventional radiography techniques and cone beam computed tomography (CBCT).The patient had recurrent throbbing pain in her # 46. The radiographic examination including “panoramic radiography and CBCT” revealed that radiographic evidence of internal resorption in #37 #36 #35 #34 #33 #47 #46 #45 #44 #43 #16 #15 #14 #13 and also including in unerupted #17, #26, #27, #28 teeth. The definitive diagnosis was made with the histopathological examination of the extracted tooth.

**Conclusions:**

Internal root resorption is a rare clinical process that should be examined using different radiographic modalities. CBCT seems to be useful in evaluation of the lesions with superior diagnostic performance.

## Background

Resorption is either a physologic or pathologic condition which can affect hard tissues, such as bone and dental hard tissues [1] and also, can involve soft tissues and foreign materials such as necrotic pulp tissue or materials used in pulp capping or root filling extruded through the apical foramen [2].

Although resorption of primary teeth is relatively well-characterized example of physiological resorption which contains no microbiological component, root resorption in the permanent dentition is a pathologic event, does not occur naturally and might be broadly classified into external or internal resorption, based on the location of the resorption in relation to the surface [3]. When compared with external resorption, internal resorption is a rare condition in permanent teeth and its etiology and pathogenesis have not been completely elucidated. When there is no specific cause, resorption occurs as an idiopathic dystrophic change. The term of idiopathic resorption, which is used when no definitive cause can be detected, reflects our limited understanding on the causative factors of this pathological process [4].

Correct diagnosis is essential, since internal and external resorption are totally different pathological process, with different etiological factors and treatment protocols. Internal root resorption poses diagnostic concerns to the clinician because its often confused with external cervical resorption. The misdiagnosis of internal root resorption can be due to the difficulty in diagnosis of the condition [5].

Radiographically, internal root resorption appears as a fairly uniform, symmetrical or eccentric, round-to-oval radiolucent enlargement of the root canal or the pulp chamber. The margins are sharp, smooth and clearly defined, with distortion of the original root canal outline. The size and location of the resorptive defect can vary considerably [6]. One of the major problems with diagnosing and predictably managing internal and external cervical root resorption is that intraoral radiographs only reveal limited diagnostic information [7,8]. The amount of information gained from these analogue and digital periapical radiographs is incomplete due to the fact that the three-dimensional anatomy of the area being radiographed is compressed into a two-dimensional image or shadowgraph [9]. Although intraoral radiography is reasonably accurate in correctly diagnosing internal and external cervical root resorption, cone beam computed tomography (CBCT) scans results more accurate in diagnosis of the presence and type of root resorption [9].

Based on available knowledge, the unusual feature of this case that is the presence of multiple internal resorption in permanent molars, has not previously been reported in the literature. The aim of this case report is to describe a rare clinical case of multiple idiopathic internal resorption in the permanent maxillary and mandibular molars using CBCT.

## Case presentation

A 33-year-old female was referred to outpatient clinic, for the treatment of # 46 that had been causing recurrent throbbing pain. There was no history of trauma, hospitalization or medical endocrine and systemic disease. Haematological investigations including complete blood count as well as calcium, phosphorus and alkaline phosphatase were within the normal range. The head and neck examination revealed no evidence of adenopathy, paresthesia or motor nerve deficiency. Intra-oral examination revealed unerupted maxillary left molars and #17. Clinical examiantion revealed a moderate oral hygine and healthy gingival tissues. Mobility of the teeth were within normal range.

**Figure 1:**
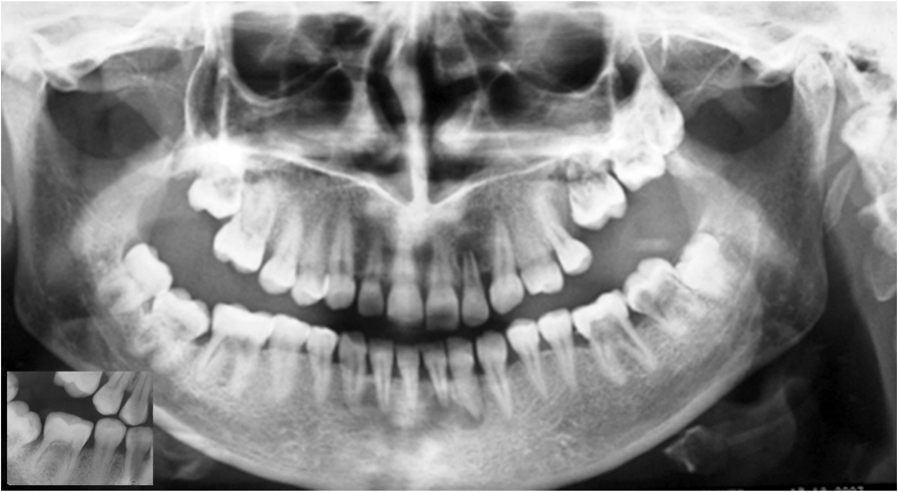
Panoramic and intraoral radiographs of the patient showing multiple idiopathic internal root resorptions.

Initially the orthopantomogram (OPG) which was taken in a local dental clinic was investigated for patient’s symptom. The radiograph showed that radiographic evidence of internal resorption in #16 #36 #37 #46 #47 and internal resorption with unerupted #17, #26, #27, #28 teeth (Figure 1).

**Figure 2:**
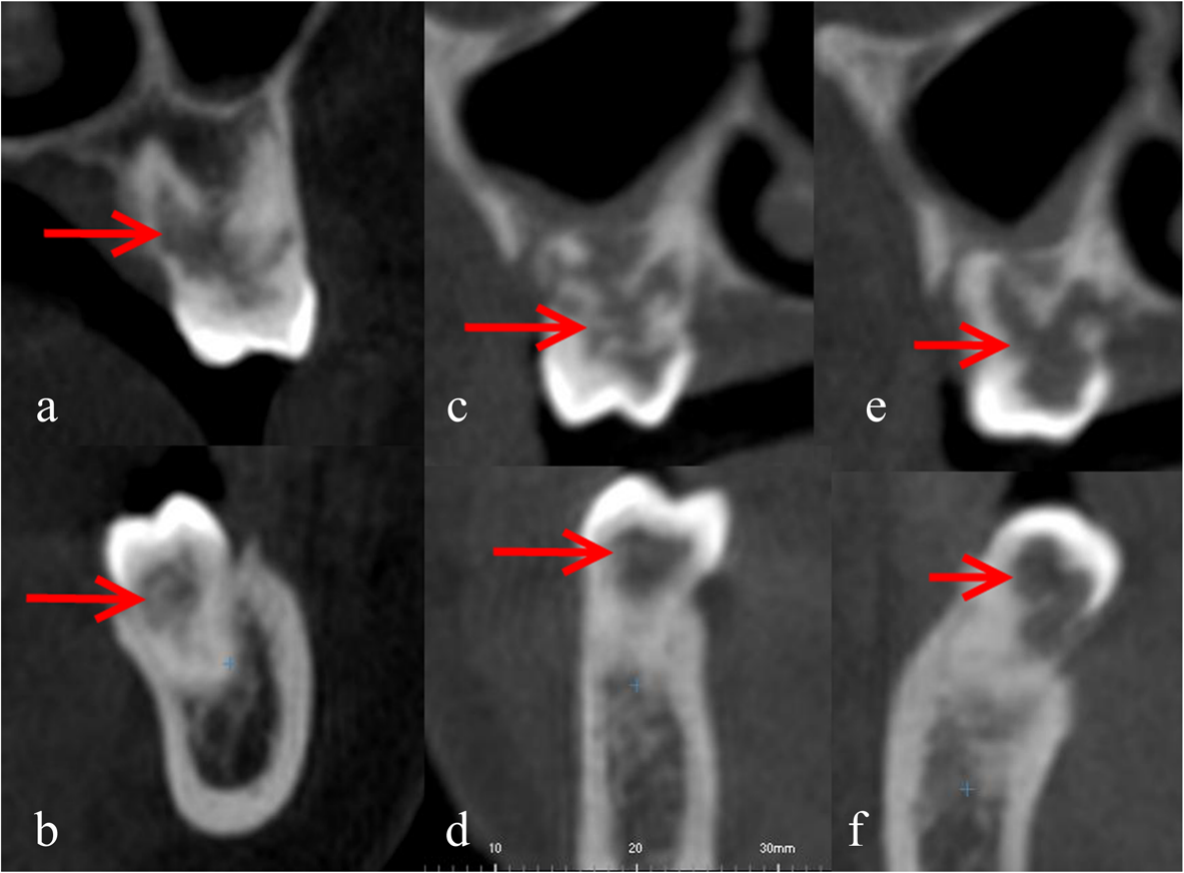
Cross sectional CBCT images showed clearly internal resorption in the pulp chambers (a: #16, b: #46, c, d: #26 e: #36, f: #37).

**Figure 3:**
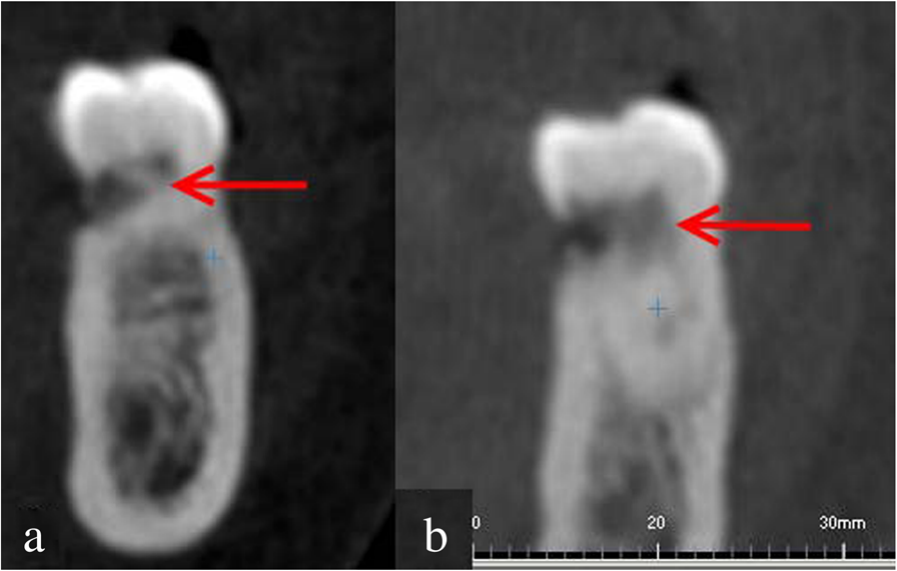
Cross sectional CBCT images showing the perforations to the lingual surfaces of #36 #37 caused by internal resorption (a: #36, b: #37).

**Figure 4:**
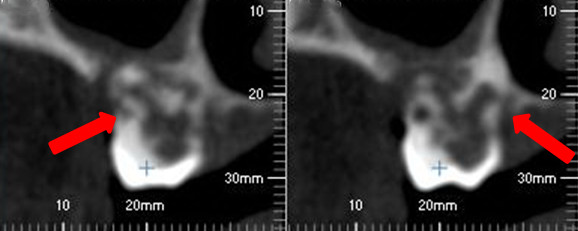
Thick maxillary sinus membrane and communication between maxillary sinus and the roots of #26 and internal resorption (arrows).

**Figure 5:**
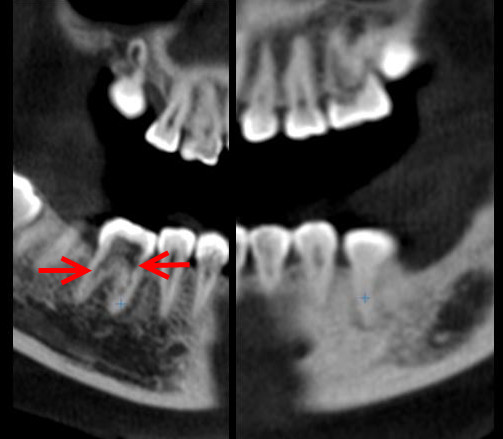
Sagittal CBCT image showing also showing clearly the internal resorption of #46.

**Figure 6:**
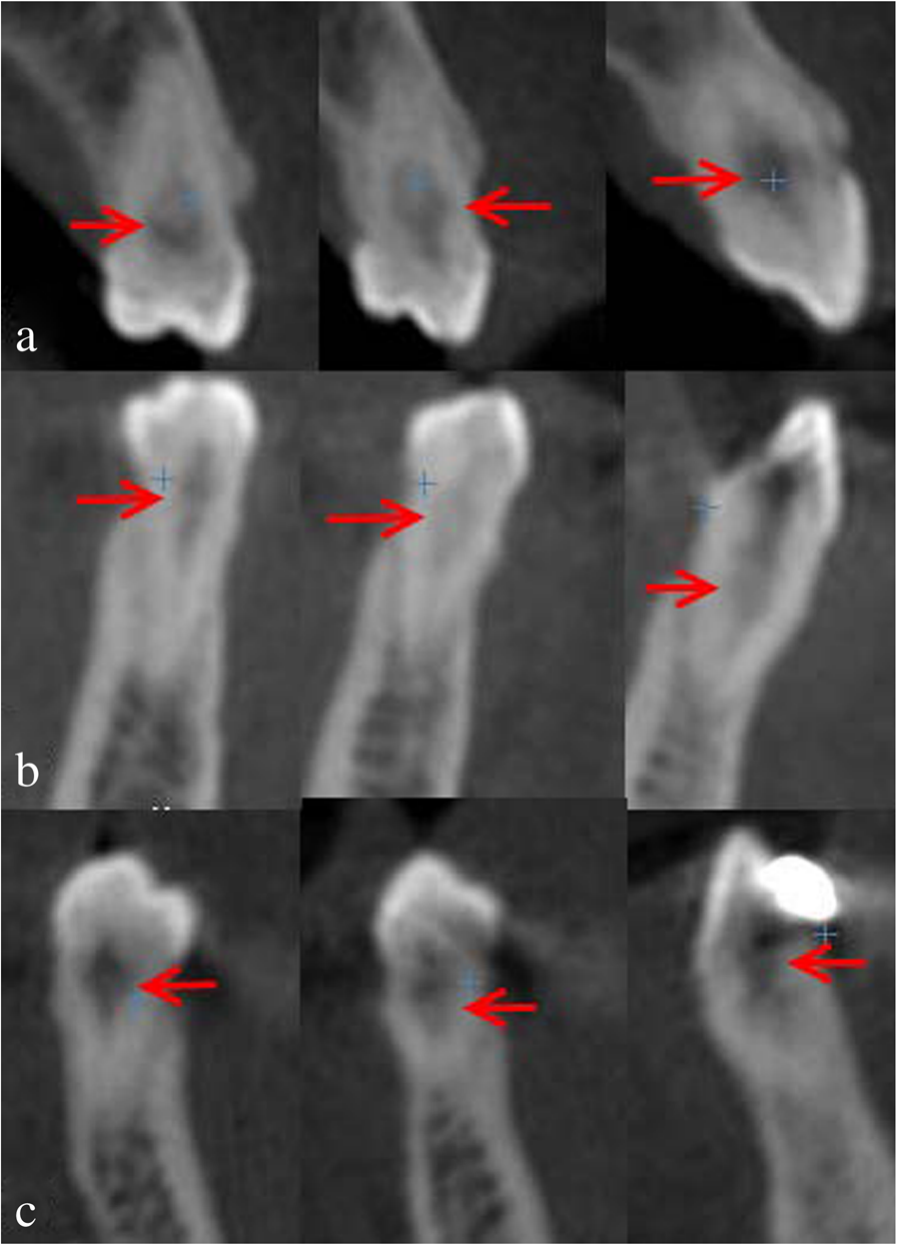
Cross-sectional CBCT image showing (a) right maxillary premolar-canine, (b) right, and (c) left mandibular canine-premlolar teeth the internal resorptions.

A decision was made to perform CBCT with three-dimensional (3D) reconstruction to obtain a more precise location and definition of the pathologic features of the resorption sites. CBCT analysis was performed in all three dimensions - axial, sagittal and cross-sectional images - with a 0.5-mm slice thickness (Iluma Ultra Cone Beam CT Scanner (3M ESPE, St. Paul, USA) together with 3D rendered images. CBCT images showed clearly internal resorption in the pulp chamber of the teeth (Figure 2). It was also diagnosed that the internal resorption caused perforations in the teeth such as a perforation to the lingual surfaces of #36 #37 (Figure 3). Besides, cross-sectional CBCT images of #26 showed an apical periodontitis which initated thicknening of maxillary sinus membrane became thick together with a communication between maxillary sinus and the roots of #26 (Figure 4). Moreover, based on patient’s complaints, #46 was examined separately. The tooth was positive on electric pulp testing, as were the other teeth in the quadrant and exhibited neither caries nor discoloration, and was slightly tender to percussion. CBCT images showed also the internal resorption in distal root and the pulp chamber and a perforation of the resorption to lingual surface of the tooth (Figures 3 and 5). Moreover, while examing CBCT cross sectional images, it was also demonstrated multiple resorption in #15 #14 #13 #33 #34 #35 #43 #44 #45. The internal resorptions in these teeth were not clear in OPG images and some of them even couldn’t visualize in this image. CBCT clearly demonstrate the interal resorption cavities in these teeth as well (Figure 6).

Based on the multiple internal resorption diagnosis on OPG and CBCT images, it was also decided to do a dental examination for the other family members to detect multiple internal resorptions because of the hereditary influence. However, no clinical or radiographic examination could be performed, as it was not possible to contact with any of the family members who were all living in another city.

Firstly, it was decided to initiate a root canal treatment for #46 and attempt to repair the resorptive defect using mineral trioxide aggregate (MTA) because of the major complaint of the patient.

When a perforation area was detected on the lingual surface of the crown after the coronal access was carried out. The whole lesion was completely debrided and irrigated, and mineral trioxide aggregate (MTA) (ProRoot, Dentsply/Tulsa Dental, Tulsa, OK) was applied and compacted into the defect before it was temporarily sealed. However, at the end of the 2 weeks, a draining sinus was constituted on the buccal surface. Thus, the tooth was decided to be extracted because the infection was observed due to the areas of internal resorption opened to oral environment.

Although it was decided to continue the treatment of all other teeth with internal resorption after the main complaints associated with #46 is over, the patient did not attend her subsequent appointments.

**Figure 7:**
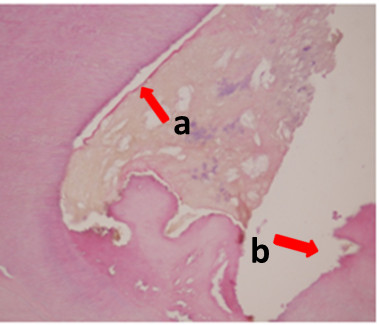
Histopathologic image showing (a) chronic inflammation with wide areas of lympho-plasma cell infiltration and epithelial cell proliferation in the pulp tissue, (b) generalized resorption and areas of new osteodentin formation in dentin.

The extracted tooth was examined histologically and the pulp tissue showed chronic inflammation with wide areas of lympho-plasma cell infiltration and epithelial cell proliferation. Dentin showed generalized resorption and areas of new osteodentin formation. It was assumed that the epithelial cells came from outside of the teeth after internal resorption reached root perforation (Figure 7).

Multiple internal resorption is rare and its etiology is unknown, although several predisposing factors have been implicated: carious, infection of the pulp, pulpal exposure, trauma, pulpotomy, extreme heat, orthodontic treatment and hereditary influence. These predisposing factors stimulate the pulp tissue, inflammation occurs and then some of the undifferentiated cells within the pulp may become converted to osteoclast or macrophages, resulting in dentinal resorption [10]. The relationship with systemic disease has not been reported yet [11,12].

Rabinowitch [13] reported a case of internal resorption in which nothing in the patient’s history, except bruxism that could explain the phenomenon. Urban et al. [12] noted the internal resorption process in maxillary left central incisor in twins and reported the link between interleukin (IL)-1 gene polymorphism and root resorption. Stewart [4] also reported the internal resorption process in maxillary primary incisors in twins and suggested a hereditary influence.

In our case, the patient had multiple internal resorption in her molar teeth some of which were also unerupted. This case can be classified as a true idiopathic resorption because no local or systemic factors related to the root resorption were found. The patient received no orthodontic treatment and any form of teeth bleaching. She had not suffered any orofacial region injury and there is also no record of systemic condition and genetic predisposition that could contribute to the development of these findings. During the intra-oral examination no evidence of parafunctional habits, occlusal trauma or periodontal disease was found.

Calcific changes were also reported in cases of internal resorption where no association with the surrounding bone was mentioned. Sweet [14] reviewed the literature on internal resorption and cited several cases in which reparative changes took place, and newly formed calcified tissue replaced the dentine and the pulp. Yoneda et al. [15] reported that internal resorption may result in perforation of the root surface or fracture of the tooth and granulation tissue that increased after perforation is considered to be the true cause of oral malodor. In our case granulation tissue that increased after the perforation of internal resorption was considered to be a cause of malodour.

The diagnosis of the internal resorption is primarily based on radiographic examination, with supplementary information gained from history and clinical findings. The difficulty in distinguishing internal resorption from external cervical resorption (ECR) has been highlighted in the literature. Mattar et al. [16] reported a case of external multiple invasive cervical resorption according to location and clinical and radiographical features of the lesions. The problem in diagnosis occurs when the ECR lesion is no accessible by probing and is projected radiologically over the root canal. Both lesions might have a similar radiographic appearance. Internal root resorption lesions are smooth and generally symmetrically distributed over the root. The radiolucency of the internal root resorption has uniform density and the pulp chamber or root canal outline can not be followed through the lesion, because the canal walls essentially balloon out. Internal root resorption lesions might also be oval, circumscribed radiolucencies in continuity with the canal walls, by contrast, ECR lesions have borders that are ill-defined and asymmetrical, with radiodensity variations in the body of the lesion. The canal wall should be traceable through the ECR lesion because it is superimposed over the root canal [17].

Once internal root resorption has been diagnosed, the clinician must make a decision on the prognosis of the tooth. If the tooth is deemed restorable and has a reasonable prognosis, root canal treatment is the treatment of choice. The aim of root canal treatment is to remove any remaining vital, apical tissue and the necrotic coronal portion of the pulp that might be sustaining and stimulating the resorbing cells via their blood supply, and to disinfect and obturate the root canal system [17]. On the other hand, Treatment of externak cervical root resorption depends on the severity, location, whether the defect has perforated the root canal system and the restorability of the tooth. Several treatment regimes have been suggested in the literature, depending on the nature of the external cervical root resorption lesion.This include intentional replantation, guided tissue regeneration, treating the external cervical root resorption lesion by an internal approach only, and forced orthodontic eruption. Essentially treatment involves complete removal of the resorptive tissue and restoring the defect. Endodontic treatment might also be required in cases in which the lesion has perforated the root canal [18].

Several studies concluded that the probability of false-negative results is one of the limitations of methods that use conventional radiography to diagnose inflammatory root resorption. Diagnostic accuracy based on conventional and digital radiographic examination is limited by the fact that the images produced by these techniques only provide a 2-dimensional representation of 3-dimensional objects [9]. The high accuracy of CBCT images is a valuable tool for analysis of tooth structure and adjacent anatomy [19]. Because CBCT scans provide 3-dimensional views, they present superior diagnostic performance over conventional radiographic images to determine the true extension of the resorptive process [19]. Some of the benefits of using CBCT in the diagnosis of endodontic disease are its high accuracy in detecting root lesions at the earliest stages, the support that it provides to establishing a differential diagnosis, and the fact that it is a non-invasive technique [9,20].

Several case reports and case series have confirmed the usefulness of CBCT in diagnosing and managing resorptive lesions [20]. Patel et al. [7] compared the accuracy of intraoral periapical radiography with CBCT for the detection and management of root resorption lesions.

In this case 9 molar, 6 premolar and 3 canine teeth with internal resorption were detected using intraoral radiography and CBCT images. We have chosen CBCT for better diagnosing the true extend of the lesion as well for detection of possible perforations with the lesions. The authors concluded that the superior accuracy of CBCT warrants the true nature of the lesion might be assessed, including root perforations and whether the lesion is amendable to treatment by using CBCT [17]. However, although the present case in the initial diagnosis demonstrated that CBCT images were superior in diagnostic efficacy to panoramic image, it should be reminded that CBCT images should not necessarily replace first stage conventional intra-oral periapical images. From the standpoint of radiation risk, CBCT appears to have three to seven times the risk of a panoramic examination depending on the area examined, the degree of collimation and the acquisition software version. Thus, the decision to select an imaging modality for diagnostic purposes as in this case for maxillary sinus follow-up should be based on the diagnostic yield expected, and in accordance with the ALARA (As Low As Reasonably Achievable) principle [21,22].

Although it has been reported that spontaneous arrestment of the resorptive process in primary molars may be considered as a possible outcome if an inflammatory background does not exist, several authors have suggested endodontic treatment as soon as the internal resoprtion is detected if a perforation of the outer surface and/or fracture of the tooth had not yet occurred [23]. However, different approaches exist in the treatment of a perforating internal resorption. Root canal therapy combined with surgical correction may be the only option in some cases [24]. Remineralization therapy with calcium hydroxide, which forms a hard tissue matrix against which to condense the root-filling material, has been advocated by others [25]. Application of MTA at the perforation site precluded, as in this case, the need for surgical intervention or prolonged treatment with calcium hydroxide. MTA can provide good sealing of the defect, subsequently allowing a conventional root canal-filling technique [26].

MTA can be selected because of its known abilities as a repair material, along with its sealing ability and mechanical strength. Although the MTA material resulted in rapid resolution of symptoms and signs, successful repair of perforation of internal resorption could not be seen for this particular case.

## Conclusions

In conclusion, inflammatory root resorption is a multidisciplinary problem, requiring a variety of specialists to understand the etiology, pathogenesis, diagnosis and treatment of this disease. Once idiopathic internal resorptions in several teeth are identified, thorough CBCT examination and short-term follow-up should be performed to preserve the teeth from rapid resorptive process and long-term follow-up should be conducted to examine evidence of the success of treatments performed.

## Consent

Written informed consent was obtained from the patient for publication of this case report and accompanying images. A copy of the written consent is available for review of the journal.

## Competing interests

The authors declare that they have no competing interests.

## Authors’ contributions

AK and MD participated in clinical dental care of the patient. KO prepared the CBCT images and rendering of the resorption case. AK, UA and FB was responsible for the literature search and wrote the paper. All authors read and approved the final manuscript.

## Pre-publication history

The pre-publication history for this paper can be accessed here:

http://www.biomedcentral.com/1472-6831/14/39/prepub
